# The complete chloroplast genome of *Lessertia frutescens* (L.) Goldblatt & J. C. Manning (Leguminosae), an important medicinal plant species from Southern Africa

**DOI:** 10.1080/23802359.2021.1967811

**Published:** 2021-08-25

**Authors:** Ying Guo, Hafiz Muhammad Wariss, Rong Zhang

**Affiliations:** a Yunnan Key Laboratory for Dai and Yi Medicines, Yunnan University of Chinese Medicine, Kunming, China; b Germplasm Bank of Wild Species, Kunming Institute of Botany, Chinese Academy of Sciences, Kunming, China; c Department of Botany, University of Sargodha, Pakistan

**Keywords:** *Lessertia frutescens*, chloroplast genome, IRLC, Leguminosae

## Abstract

*Lessertia frutescens* (L.) Goldblatt & J. C. Manning 2000 is an endemic species of Southern Africa with high medicinal and economic values. To facilitate exploration of its genetic resource, a complete chloroplast genome was determined using Illumina pair-end sequencing technology. The complete circular genome is 122,700 bp in length with overall 34.2% GC contents. It encodes a total of 110 genes, including 76 protein-coding genes, 30 tRNA, and four rRNA genes. The maximum-likelihood (ML) phylogenetic tree indicated that *L. frutescens* nested within the Papilionoideae and had a close relationship with *Astragalus nakaianus* and *A. mongholicus*. The newly sequenced complete chloroplast genome will help understanding the plastome evolution, genetic diversity and contribute to the genetic conservation of the natural population of *L. frutescens*.

*Lessertia frutescens* (L.) Goldblatt & J. C. Manning 2000, known as “cancer bush,” is an indigenous Southern Africa perennial shrub with attractive flowers (van Wyk and Albrecht 2008), belonging to the inverted repeat lacking clade (IRLC) of the Leguminosae (Legume Phylogeny Working Group [LPWG] 2017). It had been found to contain a large number of phytochemicals and several beneficial properties which used to treat a variety of ailments including stomach complaints, diabetes and uterine troubles. Notably, it had been used to treat and prevent cancer since 1895(van Wyk and Albrecht 2008). Traditionally, *L. frutescens* represents a variable species complex that is divided into three subspecies and several regional forms (van Wyk and Albrecht 2008). Therefore, *L*. *frutescens* requires more morphological and genetic evidence to distinguish the variable species complex and other sub-taxa. Chloroplast genome provides a powerful tool for reconstructing phylogenetic relationships and the development of molecular makers for the identification of plant species (Jansen et al. 2007; Huang et al. 2020). In the present study, the complete chloroplast genome of *L. frutescens* was studied which significantly contributed toward the phylogeny, genetics conservation and provide genetic resources for polymorphism investigations of this complex species.

The sample was collected from a cultivated individual in Kirstenbosch Botanical Garden, Cape Town, South Africa (33°57′41″S, 18°24′37″E). The voucher specimens were deposited in the herbarium of Kunming Institute of Botany, Chinese Academy of Sciences (http://www.kun.ac.cn/, Tao Deng, dengtao@mail.kib.ac.cn) under the voucher number G15518. Total genomic DNA was isolated with a modified CTAB protocol (Doyle and Doyle 1987) and stored in the −80 degree refrigerator of Germplasm Bank of Wild Species, Kunming Institute of Botany, Chinese Academy of Sciences (http://www.genobank.org/, Rong Zhang, zhangronga@mail.kib.ac.cn). Part of DNA was sent to Beijing Genomics Institute, Shenzhen, China for constructing a paired-end (PE) library and sequencing using the Illumina HiSeq2000. The chloroplast genome was assembled and annotated following Zhang et al. (2020). *Arachis hypogaea* L. (GenBank: NC026676) was selected as reference genome. Bandage Linux v.8.0 (Wick et al. 2015) was used to assemble the contigs and Bowtie2 (Langmead and Salzberg 2012) was used for mapping PE reads to the chloroplast genome. Finally, annotation was performed on GeSeq (Tillich et al. 2017), coupled with manual adjustment in Geneious v.9.1.4 (Kearse et al. 2012) and the chloroplast genome with accession number MF286764 was submitted to GenBank.

The complete chloroplast genome of *L. frutescens* was 122,700 bp in length which lacked the inverted repeat structure. A total of 110 genes were encoded, including 76 protein-coding genes, 30 tRNAs and four rRNAs. Similar to other IRLC chloroplast genomes, *L. frutescens* lost *rpl22* and *rps16* gene. Besides, its *clpP* gene lost the second intron, *atpF* and *rps12* genes lost an intron. The overall GC content was 34.2% while the protein-coding regions (PCRs) were 65, with 670 bp in length and 53.5% of the total chloroplast genome. The percentage of PCRs was slightly higher than that of basal taxon *Glycyrrhiza glabra* L. (NC_024038, 66,600/127,943 bp = 52.1%) of the IRLC.

To compare the topology with limited samples at subfamily level to the result with extensive samples in Zhang et al. (2020), a phylogenetic tree was constructed using RAxML v.8.2.12 (Stamatakis 2014) based on 16 plastomes representing the all six subfamilies of Leguminosae and closely related species of *L*. *frutescens*. *Quillaja saponaria* Molina was used as outgroup. The phylogenetic result (Figure 1) showed that *L*. *frutescens* nested within the subfamily Papilionoideae and formed a clade with *Astragalus nakaianus* and *A. mongholicus* with 100% bootstrap support. The subfamily Detarioideae was sister to other legumes with moderate support (57%), consistent with earlier studies based on coding genes from Zhang et al. (2020). The reconstructed phylogeny also provided robust phylogenetic relationships among four other subfamilies of Leguminosae. The newly sequenced complete chloroplast genome will provide resources for phylogenetic reconstruction of the legume family and conservation of this important medicinal plant species.

**Figure 1.**
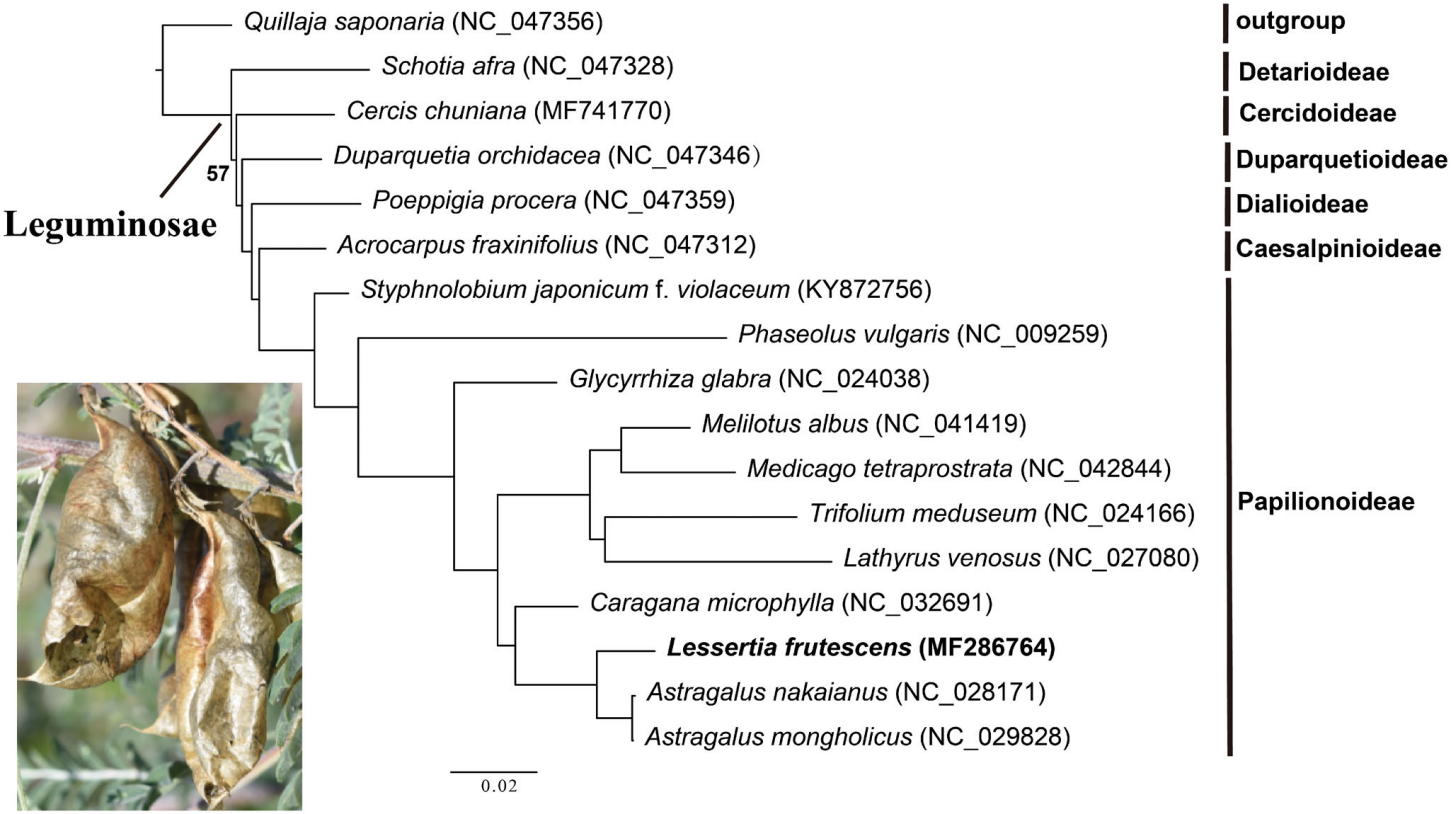
Maximum likelihood (ML) phylogenetic tree based on 17 chloroplast genomes. ML bootstrap values <100% are shown. The position of the newly sequenced *Lessertia frutescens* is shown in bold. The photograph was taken during a field collection of *L. frutescens*.

## Data Availability

The genome sequence data of *L. frutescens* are openly available in GenBank of NCBI [https://www.ncbi.nlm.nih.gov] (https://www.ncbi.nlm.nih.gov/) under the accession no. MF286764, the associated BioProject, SRA, and Bio-Sample numbers are PRJNA739544, SRR14868908, and SAMN19791851 respectively.
